# Immune selection in neoplasia: towards a microevolutionary model of cancer development

**DOI:** 10.1054/bjoc.2000.1206

**Published:** 2000-05-18

**Authors:** S J Pettit, K Seymour, E O'Flaherty, J A Kirby

**Affiliations:** Surgical Immunobiology Group, Department of Surgery, University of Newcastle upon Tyne, NE2 4HH, UK

**Keywords:** immune selection, evolution, tumour, immune escape

## Abstract

The dual properties of genetic instability and clonal expansion allow the development of a tumour to occur in a microevolutionary fashion. A broad range of pressures are exerted upon a tumour during neoplastic development. Such pressures are responsible for the selection of adaptations which provide a growth or survival advantage to the tumour. The nature of such selective pressures is implied in the phenotype of tumours that have undergone selection. We have reviewed a range of immunologically relevant adaptations that are frequently exhibited by common tumours. Many of these have the potential to function as mechanisms of immune response evasion by the tumour. Thus, such adaptations provide evidence for both the existence of immune surveillance, and the concept of immune selection in neoplastic development. This line of reasoning is supported by experimental evidence from murine models of immune involvement in neoplastic development. The process of immune selection has serious implications for the development of clinical immunotherapeutic strategies and our understanding of current in vivo models of tumour immunotherapy. © 2000 Cancer Research Campaign


*British Journal of Cancer* (2000) **82**(12), 1900–1906
© 2000 Cancer Research Campaign
doi: 10.1054/ bjoc.2000.1206, available online at http://www.idealibrary.com on IDEAL®

Review

### Immune selection in neoplasia: towards a microevolutionary model of cancer development

**SJ Pettit, K Seymour, E O'Flaherty and JA Kirby**

Surgical Immunobiology Group, Department of Surgery, University of Newcastle upon Tyne, NE2 4HH, UK

**Summary** The dual properties of genetic instability and clonal expansion allow the development of a tumour to occur in a microevolutionary fashion. A broad range of pressures are exerted upon a tumour during neoplastic development. Such pressures are responsible for the selection of adaptations which provide a growth or survival advantage to the tumour. The nature of such selective pressures is implied in the phenotype of tumours that have undergone selection. We have reviewed a range of immunologically relevant adaptations that are frequently exhibited by common tumours. Many of these have the potential to function as mechanisms of immune response evasion by the tumour. Thus, such adaptations provide evidence for both the existence of immune surveillance, and the concept of immune selection in neoplastic development. This line of reasoning is supported by experimental evidence from murine models of immune involvement in neoplastic development. The process of immune selection has serious implications for the development of clinical immunotherapeutic strategies and our understanding of current in vivo models of tumour immunotherapy. © 2000 Cancer Research Campaign

**Keywords:** immune selection; evolution; tumour; immune escape

It is widely accepted that cancer results from the accumulation of mutations in genes responsible for control of cell survival and cell death. Genetic analysis of human tumours at presentation reveals a striking degree of heterogeneity; tumours are never composed of genetically identical cells, and no two tumours are alike (Fey and Tobler, 1996). A recent review strongly supports the existence of genetic instability, at both nucleotide and chromosome level, as the driving force behind the accumulation of mutations in nearly all solid tumours (Lengauer et al, 1998). This combination of genetic instability and clonal expansion allows a process of selection to occur within a tumour, in which subclones with a survival or growth advantage will come to predominate the tumour in time.

In recent years a broad range of tumour-associated antigens (TAA), restricted by both class I and class II MHC molecules, have been identified. This has been facilitated by techniques using recombinant DNA technology, cellular immunology, and serological screening with autologous antibodies. Human TAAs have been identified in a variety of tumours and are likely to represent a small proportion of potential antigens expressed. Such TAAs include:

- Tumour-specific shared antigens such as members of the MAGE family, encoded by normal, non-mutated genes, though restricted in expression to tumours and immune privilege sites such as the testis.
- Tissue-specific differentiation antigens such as tyrosinase.
- Tumour specific antigens generated by point mutations or translocations.
- Ubiquitous antigens with a significant degree of over-expression in tumours (Rosenberg, 1999).

*Received 13 April 1999*
*Revised 1 February 2000*
*Accepted 17 February 2000*

*Correspondence to:* SJ Pettit

There is currently little consensus on the extent to which tumour and host immune system interact. It is becoming clear that the immune response has evolved to be activated by recognition of pathogen-associated molecular patterns (PAMPs) within an inflammatory context (Janeway et al, 1996). It is difficult to envisage how an adaptive anti-tumour immune response would be initiated. The majority of human tumours do not express PAMPs and tumorigenesis is initially an innocuous extension of self. Moreover, examination of patients with more advanced tumours shows little evidence of an effective host response. These considerations have led many immunologists to conclude that while tumours may be antigenic, they are unlikely to be immunogenic. As such, an immunological ignorance or a progressive tolerance would be expected to occur during the natural history of a tumour.

Interestingly, several studies have identified mechanisms by which tumour cells may potentially evade an immune response. This review will examine the pattern and frequency with which 'immune escape' mechanisms occur in human tumours. The nature of each immune escape mechanism will be assessed in the context of known or potential interactions between tumour and host immune system. A microevolutionary model of tumour development involving escape from immunological control will then be presented. The implications of this model for the development of clinical immunotherapeutic strategies and our understanding of current in vivo models of tumour immunotherapy will be discussed.

### Anti-tumour immunity and immune escape mechanisms

The identification of a broad range of TAAs provides the basis for specific rejection of tumours by the cell-mediated arm of the adaptive immune system. Many studies identifying TAAs have used tumour-specific $CD8^+$ and $CD4^+$ T cell clones raised from the autologous host (Boon, 1992; Cox et al, 1994). While these studies demonstrate the existence of tumour-specific T cells in cancer patients, they provide no evidence for a functional role of these T cells in anti-tumour immune responses. However, studies have identified TAAs by use of circulating tumour-specific antibodies in the autologous host; such TAAs may also be recognized by CTL (Chen et al, 1997). The identification of a variety of circulating tumour-specific antibodies in the serum of cancer patients suggests that tumours are capable of eliciting multiple specific immune responses (Sahin et al, 1995).

The presence of tumour-infiltrating lymphocytes (TIL) has been demonstrated in biopsies of a variety of histological types of tumour. Isolation and characterization of TIL has shown that they constitute both $CD4^+$ and $CD8^+$ $\alpha\beta$ T cells that may recognize autologous tumours and tumour cell lines in assays of cytotoxicity and cytokine secretion. However, the nature of TIL depends on the tumour type from which they were derived. Melanoma-derived TIL exert a greater anti-tumour effect than those derived from breast or colon cancer biopsies (Yannelli et al, 1996). Early studies using MHC-peptide tetramer staining showed high numbers of antigen-experienced CTL specific for Melan-A in metastatic lymph nodes of melanoma patients. These CTL were capable of clonal expansion and autologous tumour lysis (Romero et al, 1998).

The classical MHC class I pathway functions to present largely endogenous peptides to CTL, and is understood to restrict the anti-tumour T cell response. Alteration of MHC class I expression is a widespread occurrence in neoplasia, though irregular in pattern (Garrido et al, 1993). It may take the form of a complete loss of MHC class I expression, or more frequently, a partial MHC class I expression involving loss of haplotypes, loci, or individual alleles. Reduction in expression of the MHC class I element which restricts tumour-specific CTL cytotoxicity may reflect a process of immune escape during the development of the tumour.

MHC class I downregulation may result from structural defects in MHC genes, downregulation of MHC gene transcription, defects in $\beta_2$-microglobulin ($\beta_2$m) synthesis, or defects in MHC molecule assembly. Peptide fragments are usually generated by the multicatalytic proteosome complex, particularly LMP-2 and -7 subunits. These subunits may alter the pattern of protein cleavage within the proteosome, favouring the generation of immunogenic peptides (Driscoll et al, 1993). Peptides are subsequently transported into the endoplasmic reticulum (ER) by a complex consisting of transporters associated with antigen presentation, TAP1 and TAP2 subunits. Within the ER, peptide binds to the heavy chain and $\beta_2$ m to form a stable peptide-MHC molecule complex (York and Rock, 1996). It is known that defects in this antigen processing and presentation machinery impair the assembly of class I MHC molecules, and thus decrease their cell surface expression and stability (Ljunggren et al, 1990). Analysis of small cell lung carcinoma (Restifo et al, 1993), hepatocellular carcinoma (Kurokohchi et al, 1996), melanoma (Maeurer et al, 1996) and renal cell carcinoma (Seliger et al, 1996*a*; 1996*b*) lines has revealed a heterogeneous downregulation of LMP-2, LMP-7, TAP-1 and TAP-2.

Natural killer (NK) cell surveillance is believed to participate in anti-tumour immunity. NK cell cytotoxicity is controlled by a balance of triggering and inhibitory signals. Inhibitory signals are received through engagement of killer inhibitory receptors (KIRs) with specific MHC class I molecules on target cells (Lanier, 1998). Reduction in MHC class I expression to decrease susceptibility to CTL lysis may therefore increase susceptibility to NK cell cytotoxicity. Alterations in MHC class I expression for purpose of immune escape would be expected to reflect this dual pressure. This is supported by data from cervical cancer patients which demonstrate that downregultion of MHC class I expression is accompanied by a relative over-representation of alleles specific for the individual patient KIRs (Keating et al, 1995). Immune escape from NK cell cytotoxicity is concordant with the frequent loss of MHC class I haplotype, locus, or allele expression, rather than the complete loss of MHC class I expression (Garrido et al, 1997).

CTL and NK cells induce apoptosis in target cells by activation of intracellular caspase cascades. Expression of *Fas* ligand (*Fas*L) or membrane TNF allows direct ligation of *Fas* or TNF receptors, constitutively expressed on the surface of most cell types, and transduction of a signal which activates intracellular caspases. Degranulation of CTL and NK cells leads to perforin-mediated pore formation and cytoplasmic introduction of granzyme B, a protein capable of direct activation of intracellular caspase-8 (Berke, 1995; Medema et al, 1997). Tumours are frequently observed to lose sensitivity to apoptosis. Blockade of the intracellular transduction of apoptotic signals has been demonstrated to occur in melanoma by expression of FLICE-like inhibitory proteins that inhibit activation of caspase-8 (Irmler et al, 1997). Overexpression of the anti-apoptotic proteins Bcl-2 or Bcl-$X_L$ has been observed in many tumours, including head and neck tumours (Pena et al, 1999), gastric carcinoma (Aizawa et al, 1999), and oesophageal carcinoma (Torzewski et al, 1998). Alteration of sphingomyelinase activation and subsequent ceramide generation has been suggested to function in preventing TNF-mediated cytotoxicity in breast tumours (Cai et al, 1997).

Such adaptations are intrinsic to tumour growth. However, tumours also exhibit mechanisms to block the initial extracellular signals provided by T or NK cells for the induction of apoptosis. The genomic amplification and secretion of decoy receptor 3 (DcR3) in colon, lung, breast and gastric tumours was recently described (Pitti et al, 1998). DcR3 specifically binds and blocks the function of *Fas*L. The secretion of soluble *Fas* (s*Fas*) has also been observed in hepatocellular carcinoma (Jodo et al, 1998). In a similar fashion to DcR3, s*Fas* binds and blocks the function of *Fas*L (Cheng et al, 1994), thus inhibiting T cell and NK cell cytotoxicity. Such mechanisms may allow effective escape from immunological control.

There is a large body of evidence to support the view that stimulation of apoptotic cell death by the *Fas*-*Fas*L pathway may also be reversed in the host-tumour immune interaction. The expression of *Fas*L has been identified in melanoma (Hahne et al, 1996), colon carcinoma (O'Connell et al, 1998), hepatocellular carcinoma (Strand et al, 1996), gastric adenocarcinoma (Bennett et al, 1999) and lung carcinoma (Niehans et al, 1997). In a process termed the '*Fas* counterattack', it is suggested that tumour cells might be able to promote the death of activated tumour-specific T cells or NK cells within the cancer microenvironment, thus creating a tumour-specific window of tolerance. Such tumours frequently lose expression of *Fas* (Strand et al, 1996), ostensibly to avoid the induction of suicidal or fratricidal cell death, though insensitivity to apoptosis might allow coexpression of *Fas* and *Fas* L. The development of surface *Fas*L expression by a tumour cell would constitute an effective mechanism of immune escape.

Tumours have been observed to secrete a range of cytokines with the potential to inhibit activation of, downregulate or subvert

*© 2000 Cancer Research Campaign* 

the host immune response. Secretion of immunosuppressive cytokines into the immediate microenvironment may reflect a process of immune escape. TGF-β is a potent immunosuppressive factor, and affects activation, proliferation and differentiation of both innate and adaptive immune cells (Chouaib et al, 1997). Studies have identified expression and secretion of TGF-β in bladder tumours (Eder et al, 1997), gastric carcinoma (Morisaki et al, 1996), breast and hepatocellular carcinoma (Vanky et al, 1997). Both TGF-β and IL-10 are understood to play a role in shifting the Th1-Th2 balance toward Th2, thus inhibiting the cell-mediated immunity believed to be responsible for tumour rejection (Maeda and Shiraishi, 1996). Secretion of IL-10 has been observed in renal cell carcinoma, colon carcinoma, melanoma and neuroblastoma (Gastl et al, 1993). Studies have demonstrated that a shift toward a Th2 cytokine profile can inhibit an otherwise effective anti-tumour immune response (Hu et al, 1998). Furthermore, it has been demonstrated that local IL-10 secretion by tumours can confer a complete resistance to CTL lysis (Matsuda et al, 1994).

Adhesive interactions between immune cells and target cells are critical to the processes of recognition and cytotoxicity. Alterations in the expression and function of intercellular adhesion molecules are frequently observed in tumours. The capacity of a tumour cell to inhibit adhesive interactions with immune effector cells may represent a potentially effective mechanism of immune escape. It is becoming clear that the mucosal immune response is mediated by a subset of T cells expressing $\alpha^E\beta7$-integrin. Such T cells are restricted by adhesion through $\alpha^E\beta7$-integrin to E-cadherin expressing epithelial target cells (Karecla et al, 1996). The loss of E-cadherin expression on epithelioid tumours would allow effective escape from immunological control by $\alpha^E\beta7$-integrin-dependent T cells. Studies in pancreatic cancer have demonstrated loss of E-cadherin expression after infiltration with $\alpha^E\beta7$-integrin-expressing T cells, associated with the progression of a well differentiated tumour to a poorly differentiated and more invasive phenotype (Ademmer et al, 1998). it is understood that loss of E-cadherin expression leads to a breakdown in cell–cell adhesion and activation of several signalling pathways (Christofori and Semb, 1999). As such, loss of E-cadherin expression for purpose of immune escape may incidentally lead to the development of an aggressive, pro-metastatic tumour phenotype.

Several epitheloid tumours including bladder, breast, ovarian and colorectal carcinomas are known to alter their expression pattern of large mucin molecules such as MUC-1. Such molecules project from the cell surface and may interfere with normal intercellular adhesion by steric blockade of the molecular interactions (Taylor Papadimitriou and Finn, 1997). Several groups have shown that the expression of MUC-1 can protect tumour cells from cytolysis by CTL (van de Welvankemenade et al, 1993) and NK cells (Zhang et al, 1997). It has also been reported that MUC-1 can inhibit proliferation and induce an anergic state in T cells (Agrawal et al, 1998). Such interactions suggest that MUC-1 expression may constitute another effective mechanism of immune escape.

The nature of these adaptations, when considered together, provides compelling evidence for a process of escape from immunological control in many common human tumours. This is supported by functional analysis of TAA-specific elements of the adaptive immune system in patients with advanced cancer. One such study used MHC-peptide tetramer staining to isolate circulating $CD8^+$T cell populations specific for MART-1 and tyrosinase TAAs from melanoma patients. It was demonstrated that TAA-specific CTL have been rendered functionally unresponsive in vivo; isolated CTL were incapable of antigen-specific target cell lysis or cytokine production (Lee et al, 1999).

### Model of immune selection in neoplasia

Complex adaptations exhibited by tumours are unlikely to be developed at random. The combination of genetic instability and clonal expansion allows a gradual process of microevolution to occur, in which subclones with a survival or growth advantage come to predominate the tumour. This requires selective pressure. Competition for space, ability to recruit neovasculature and the ability to detach from neighbouring cells may all represent early selective pressures. Microevolutionary tumour development has long been recognized in the field of chemotherapy for neoplasia. Following drug-induced regression, approximately 30% of tumours recur in a form resistant to the chemotherapeutic agent (Young, 1989). This process is termed acquired drug resistance.

It has been established that a variety of immune escape mechanisms are commonly expressed by tumours of diverse histological type. Although widely acknowledged, the scientific community has not focused on the implication for immunological involvement in tumour development. Mechanisms of immune escape are unlikely to occur spontaneously in such a consistent fashion. Rather, it may be argued that immune escape mechanisms reflect a process of microevolution following a period of sustained selective pressure by an anti-tumour host immune response.

It is unclear how the immune system would survey for neoplasia. Activation of an adaptive response would require processing and presentation of tumour antigen to tumour-specific T cells by professional APCs. The majority of human tumours do not express PAMPs. Moreover, the initial period of tumour growth is an innocuous extension of self and generally not associated with any ‘danger’ signals (Fuchs and Matzinger, 1996). Thus, tumours would not be expected to stimulate APC maturation and the activation of an anti-tumour adaptive response. The host would remain immunologically ignorant, and thus completely tolerant to the tumour, over the initial years of tumour development. A recent study showed this type of immunological ignorance to solid tumours in vivo (Ochsenbein et al, 1999). In this study, tumour growth correlated with the failure of tumour antigen to reach local lymph nodes and the absence of primed CTL. Importantly though, this study demonstrated that established solid tumours could be rejected by effective induction of an anti-tumour T cell response.

Progressive tumour growth may eventually generate signals capable of stimulating an adaptive response. An inflammatory reaction commonly occurs within the tumour microenvironment (Hakansson et al, 1997). Ischaemia and oxidative stress within the tumour microenvironment may lead to the upregulation of heat shock protein expression, identified as potential danger signals (Multhoff et al, 1995). Necrotic cell death, demonstrated to be highly immunogenic, frequently occurs at the tumour core (Melcher et al, 1998). It is reasonable to expect that such signals will activate tumour-resident professional APCs and allow presentation of TAAs to T cells (Gallucci et al, 1999). This is supported by studies that have demonstrated activation of the immune system by heat shock proteins expressed by tumours (Todryk et al, 1999) and the constitutive processing and presentation of tumour antigen in lymph nodes draining tumour sites (Marzo et al, 1999).

*© 2000 Cancer Research Campaign*

Activation of an anti-tumour immune response would place a novel selective pressure on established tumours. Certain subclones within the heterogeneous tumour may express gene products or exhibit characteristics that reduce the susceptibility of that subclone to an immune response. The pressure of an immune response will place such subclones at a selective advantage over other subclones within the tumour. Thus, subclones resistant to an immune response will gradually come to predominate within the tumour. Adaptations of great complexity may arise by a series of single selective events, directed by nonrandom survival, in a deceptively simple process termed cumulative selection (Dawkins, 1988). This provides a rational explanation for the development of immune escape mechanisms. Tumours subjected to immune selection would be expected to exhibit a range of immune escape adaptations of considerable sophistication at all levels of host–tumour immune interaction. This has been demonstrated in a wide variety of human tumours.

In summary, the process of immune selection may be considered to occur in three non-discrete phases, as depicted in Figure 1. During the first phase, initiation, proliferation and diversification of tumour occurs against a background of immune ignorance. The second phase is entered where an anti-tumour immune response is activated. Consequent immune selection pressure allows a process of microevolution to lead to the gradual acquisition of adaptations that permit immune evasion. The third phase of immune escape is reached when tumour cells acquire sufficient adaptations to allow proliferation unchecked by immunological control.

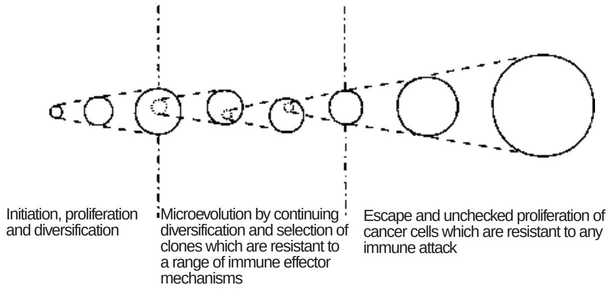

**Figure 1** Scheme of immune selection in the development of cancer. The first phase involves initiation, early proliferation and diversification of a neoplasm. The second phase involves gradual microevolution of the neoplasm under the pressure of immune selection. The third phase involves escape of the neoplasm from immunological control and unchecked progression.

### Evidence for a process of immune selection

It is difficult to provide direct evidence to support a process of immune selection and the microevolutionary development of immune escape adaptations. However, it has been possible to demonstrate the progressive nature of development of certain adaptations that may function in immune escape. While such adaptations only constitute circumstantial evidence of immune selection, their pattern of acquisition is instructive in examining how the process of immune selection might function in vivo.

Several studies have elegantly demonstrated the gradual nature of MHC class I loss with tumour progression. This process was illustrated in a study showing progressive allelic MHC class I loss in metastases of a single melanoma patient examined 5 years apart (Lehmann et al, 1995). Further evidence for this type of process was provided in a study of cervical carcinoma patients showing MHC class I downregulation in metastases compared with the primary tumour (Cromme et al, 1994). Interestingly, a recent review of alteration of TAP-1 expression in tumour biopsies in situ identified an association between the severity of abnormalities in antigen processing machinery and the progression of neoplastic disease (Seliger et al, 1997). Progressive downregulation of LMP and TAP protein expression has been demonstrated in the metastases of patients with renal cell carcinoma (Seliger et al, 1996*a*; 1996*b*). Progressive acquisition of immune escape mechanisms is consistent with the proposal of a gradual process of immune selection during the natural history of the tumour.

There is little experimental evidence on the extent of immune involvement in the development of neoplasia. Such evidence is difficult to obtain in humans; the period of interest is in the early asymptomatic phase of tumour development. Murine models of immune involvement in neoplasia have frequently focused on transplanted tumours. However, two studies have investigated immune involvement in the ‘natural’ development of neoplasia. They compared the immunogenicity of induced tumours from immunodeficient mice and congenic mice with a normal immune system. The first study used athymic nude BALB/c mice as a model of immunodeficiency (Svane et al, 1996), the second study used C.B-17 mice with severe combined immune deficiency (SCID) (Engel et al, 1997). In both studies, mice were treated with a potent carcinogen, MCA, and any tumours which developed were recovered. After brief propagation in vitro, tumours were transplanted into syngeneic immunocompetent mice. Tumours that developed in immunodeficient mice were shown to be rejected at a significantly greater rate than those developed in normal mice. These studies demonstrate that interaction with the acquired arm of the host immune system during the initiation and development of neoplasia has the effect of decreasing the immunogenicity of resulting tumours, reflecting a process of immune selection.

It must be stressed that while immune escape adaptations in human tumours can be viewed in the context of immune selection in the development of neoplasia, they do not provide firm evidence that immune selection takes place in vivo. Indeed, such adaptations may simply be markers of the process of dedifferentiation that accompanies tumour development and reflect the reacquisition of a ‘stem cell’ type phenotype. As such, these features may aid evasion of an immune response, without necessarily being selected by it. Further experimental evidence is required to allow this model of immune selection to be conclusively accepted or rejected. However, it is interesting to examine the implications of a hypothetical process of immune selection.

### Implications of immune selection

The process of immune selection in neoplasia and consequent development of immune escape mechanisms are consistent with the observation that there is little evidence of a clinically effective immune response in the majority of progressive tumours. However, an acceptance that induction of an anti-tumour immune response may lead to a process of immune selection has serious implications for the development and improvement of immunotherapeutic strategies.

© 2000 Cancer Research Campaign 

The success of immunotherapeutic strategies may depend on the natural history of the tumour. Tumours of higher stage may have been subjected to a greater degree of immune selection, and are thus more likely to be adapted to evade the very immune response that such therapeutic strategies attempt to induce. Early tumours are less likely to have been subjected to immune selection pressure. A recent study showed that small solid tumour fragments are ignored by the immune system, but may be rejected by an intervention that results in the induction of an anti-tumour immune response (Ochsenbein et al, 1999). Another study demonstrated that antigenic cancer cells may grow progressively in immunocompetent mice for 30 days with no evidence of T cell exhaustion or systemic anergy (Wick et al, 1997). These studies suggest immunotherapeutic strategies may be successful, when applied early in the natural history of the tumour.

It is well established that intravesical instillation of bacillus Calmette-Guerin (BCG) offers an effective therapeutic approach for treatment of superficial transitional cell carcinoma (TCC) of the urinary bladder (Alexandroff et al, 1999). The considerable success in the use of BCG therapy may be due, in part, to the early stage at which these tumours are diagnosed. Superficial TCC is commonly associated with symptoms such as haematuria that tend patients toward an early presentation. Moreover, patients with a history of superficial TCC are screened by regular cystoscopy for tumour recurrence. The early use of BCG therapy means that any immune component to the BCG-mediated anti-tumour response will be directed against a tumour which is unlikely to have been subjected to extensive immune selection.

Immunotherapeutic strategies might be improved by simultaneous efforts to reduce the genetic instability of tumours or to reduce the clonal expansion that allows fixation of genetic changes in the tumour cell progeny. This might be achieved by therapeutic modalities that exert a direct anti-proliferative effect or that inhibit proliferation through mechanisms such as inhibition of angiogenesis. Interestingly, it has been demonstrated that BCG exerts an anti-proliferative effect on several TCC lines in vitro (Jackson et al, 1994), and may induce the release of anti-angiogenic chemokines such as IP-10 upon intravesical instillation (Poppas et al, 1998). The real attraction of BCG therapy may be the highly complex and multifactorial anti-tumour response that is induced. This response involves elements of specific and non-specific anti-tumour immunity combined with direct cytotoxic and cytostatic effects. It is reasonable to propose that the non-specific action of BCG may prevent immune selection resulting from the tumour-specific actions of BCG.

The process of immune selection also provides an explanation for the frequent observation that promising immunotherapeutic strategies developed in murine models of cancer tend to translate poorly into human clinical trials. Murine models of cancer are frequently based on the inoculation of a mouse with a tumour cell line, previously propagated in vitro. The use of cell lines has a number of drawbacks. Cell lines are commonly derived from advanced tumours and may have already been subjected to a degree of immune selection in vivo. Moreover, cell lines retain the genetic instability of the parent tumour and may lose parental adaptations during in vitro propagation. Cell lines are introduced in large numbers, typically greater than $10^6$ cells, and thus present an unnaturally homogenous tumour burden to the immune system at first encounter. Such tumours lack the long period of development from the point of initiation at single-cell level, in which host-tumour immune interactions would be expected to develop. For these reasons, promising immunotherapeutic strategies developed in murine models of cancer may only reflect the inherent susceptibility of certain cell lines within the chosen experimental system.

### CONCLUSION

Immune evasion mechanisms have now been identified in the majority of common human tumours. These provide compelling evidence for the existence of selective pressure by the immune system, and thus for the initial existence of an anti-tumour immune response. This is supported by recent murine models of immune involvement in neoplastic development. Prolonged selective pressure without complete clearance might eventually result in the development of adaptations that allow a tumour to escape any effective immune response. This model concurs with the clinical observation that the immune system appears ineffective in the control of progressive cancer. Further work is clearly required to determine the nature of signals that may mediate activation of an immune response during neoplastic development. However, an understanding that tumours may have undergone a process of immune selection prior to clinical presentation has serious implications for the development and application of immunotherapeutic strategies.

### REFERENCES

Ademmer K, Ebert M, Muller Ostermeyer F, Friess H, Buchler MW, Schubert W and Malfertheiner P (1998) Effector T lymphocyte subsets in human pancreatic cancer: detection of CD8(+) CD18(+) cells and CD8(+) CD103(+) cells by multi-epitope imaging. *Clin Exp Immunol* **112**: 21–26

Agrawal B, Krantz MJ, Reddish MA and Longenecker BM (1998) Cancer-associated MUC1 mucin inhibits human T-cell proliferation, which is reversible by IL-2. *Nat Med* **4**: 43–49

Aizawa K, Ueki K, Suzuki S, Yabusaki H, Kanda T, Nishimaki T, Suzuki T and Hatakeyama K (1999) Apoptosis and Bcl-2 expression in gastric carcinomas: Correlation with clinicopathological variables, p53 expression, cell proliferation and prognosis. *International Journal of Oncology* **14**: 85–91

Alexandroff AB, Jackson AM, O'Donnell MA and James K (1999) BCG immunotherapy of bladder cancer: 20 years on. *Lancet* **353**: 1689–1694

Bennett M, O'Connell J, O'Sullivan G, Roche D, Brady C, Kelly J, Collins J and Shanahan F (1999) Expression of Fas ligand by human gastric adenocarcinomas: a potential mechanism of immune escape in stomach cancer. *Gut* **44**: 156–162

Berke G (1995) The CTLs kiss of death. *Cell* **81**: 9–12

Boon T (1992) Toward a genetic analysis of tumor rejection antigens. *Adv Cancer Res* **58**: 177–210

Cai ZZ, Bettaieb A, ElMahdani N, Legres LG, Stancou R, Masliah J and Chouaib S (1997) Alteration of the sphingomyelin/ceramide pathway is associated with resistance of human breast carcinoma MCF7 cells to tumor necrosis factor-alpha-mediated cytotoxicity. *J Biol Chem* **272**: 6918–6926

Chen YT, Scanlan MJ, Sahin U, Tureci O, Gure AO, Tsang SL, Williamson B, Stockert E, Pfreundschuh M and Old LJ (1997) A testicular antigen aberrantly expressed in human cancers detected by autologous antibody screening. *Proc Nat Acad Sci USA* **94**: 1914–1918

Cheng JH, Zhou T, Liu CD, Shapiro JP, Brauer MJ, Kiefer MC, Barr PJ and Mountz JD (1994) Protection from Fas-mediated apoptosis by a soluble form of the Fas molecule. *Science* **263**: 1759–1762

Chouaib S, Asselin Paturel C, MamiChouaib F, Caignard A and Blay JY (1997) The host-tumor immune conflict: from immunosuppression to resistance and destruction. *Immunol Today* **18**: 493–497

Christofori G and Semb H (1999) The role of the cell-adhesion molecule E-cadherin as a tumour-suppressor gene. *Trends Biochem Sci* **24**: 73–76

Cox AL, Skipper J, Chen Y, Henderson RA, Darrow TL, Shabanowitz J, Engelhard VH, Hunt DF and Slingluff CL (1994) Identification of a peptide recognized by 5 melanoma-specific human cytotoxic T cell lines. *Science* **264**: 716–719

 *© 2000 Cancer Research Campaign*

Cromme FV, Vanbommel PFJ, Walboomers JMM, Gallee MPW, Stern PL, Kenemans P, Helmerhorst TJM, Stukart MJ and Meijer C (1994) Differences in MHC and TAP-1 expression in cervical cancer lymph node metastases as compared with the primary tumours. *Br J Cancer* **69**: 1176–1181

Dawkins R (1988) *The Blind Watchmaker*. Penguin: London

Driscoll J, Brown MG, Finley D and Monaco JJ (1993) MHC linked LMP gene products specifically alter peptidase activities of the proteasome. *Nature* **365**: 262–264

Eder IE, Stenzl A, Hobisch A, Cronauer MV, Bartsch G and Klocker H (1997) Expression of transforming growth factors beta-1, beta 2 and beta 3 in human bladder carcinomas. *Br J Cancer* **75**: 1753–1760

Engel AM, Svane IM, Rygaard J and Werdelin O (1997) MCA sarcomas induced in scid mice are more immunogenic than MCA sarcomas induced in congenic, immunocompetent mice. *Scand J Immunol* **45**: 463–470

Fey MF and Tobler A (1996) Tumour heterogeneity and clonality – An old theme revisited. *Ann Oncol* **7**: 121–128

Fuchs EJ and Matzinger P (1996) Is cancer dangerous to the immune system? *Semin Immunol* **8**: 271–280

Gallucci S, Lolkema M and Matzinger P (1999) Natural adjuvants: Endogenous activators of dendritic cells. *Nat Med* **5**: 1249–1255

Garrido F, Cabrera T, Concha A, Glew S, Ruizcabello F and Stern PL (1993) Natural history of HLA expression during tumour development. *Immunol Today* **14**: 491–499

Garrido F, Ruiz Cabello F, Cabrera T, Perez Villar JJ, Lopez Botet M, Duggan Keen M and Stern PL (1997) Implications for immunosurveillance of altered HLA class I phenotypes in human tumours. *Immunol Today* **18**: 89–95

Gastl GA, Abrams JS, Nanus DM, Oosterkamp R, Silver J, Liu F, Chen M, Albino AP and Bander NH (1993) Interleukin-10 production by human carcinoma cell lines and its relationship to interleukin-6 expression. *Int J Cancer* **55**: 96–101

Hahne M, Rimoldi D, Schroter M, Romero P, Schreier M, French LE, Schneider P, Bornand T, Fontana A, Lienard D, Cerottini JC and Tschopp J (1996) Melanoma cell expression of Fas(Apo-1/CD95) ligand: Implications for tumor immune escape. *Science* **274**: 1363–1366

Hakansson L, Adell G, Boeryd B, Sjogren F and Sjodahl R (1997) Infiltration of mononuclear inflammatory cells into primary colorectal carcinomas: An immunohistological analysis. *Br J Cancer* **75**: 374–380

Hu HM, Urba WJ and Fox BA (1998) Gene-modified tumor vaccine with therapeutic potential shifts tumor-specific T cell response from a type 2 to a type 1 cytokine profile. *J Immunol* **161**: 3033–3041

Irmler M, Thome M, Hahne M, Schneider P, Hofmann B, Steiner V, Bodmer JL, Schroter M, Burns K, Mattmann C, Rimoldi D, French LE and Tschopp J (1997) Inhibition of death receptor signals by cellular FLIP. *Nature* **388**: 190–195

Jackson AM, Alexandroff AB, Fleming D, Prescott S, Chisholm GD and James K (1994) Bacillus Calmette-Guerin (BCG) organisms directly alter the growth of bladder tumor cells. *International Journal of Oncology* **5**: 697–703

Janeway CA, Goodnow CC and Medzhitov R (1996) Immunological tolerance – danger, pathogen on the premises. *Curr Biol* **6**: 519–522

Jodo S, Kobayashi S, Nakajima Y, Matsunaga T, Nakayama N, Ogura N, Kayagaki N, Okumura K and Koike T (1998) Elevated serum levels of soluble Fas/APO-1 (CD95) in patients with hepatocellular carcinoma. *Clin Exp Immunol* **112**: 166–171

Karecla PI, Green SJ, Bowden SJ, Coadwell J and Kilshaw PJ (1996) Identification of a binding site for integrin alpha E beta(7) in the N-terminal domain of E-cadherin. *J Biol Chem* **271**: 30909–30915

Keating PJ, Cromme FV, Duggankeen M, Snijders PJF, Walboomers JMM, Hunter RD, Dyer PA and Stern PL (1995) Frequency of downregulation of individual HLA-A and HLA-B alleles in cervical carcinomas in relation to TAP-1 expression. *Br J Cancer* **72**: 405–411

Kurokohchi K, Carrington M, Mann DL, Simonis TB, Alexander Miller MA, Feinstone SM, Akatsuka T and Berzofsky JA (1996) Expression of HLA class I molecules and the transporter associated with antigen processing in hepatocellular carcinoma. *Hepatology* **23**: 1181–1188

Lanier LL (1998) NK cell receptors. *Annu Rev Immunol* **16**: 359–393

Lee PP, Yee C, Savage PA, Fong L, Brockstedt D, Weber JS, Johnson D, Swetter S, Thompson J, Greenberg PD, Roederer M and Davis MM (1999) Characterization of circulating T cells specific for tumor-associated antigens in melanoma patients. *Nat Med* **5**: 677–685

Lehmann F, Marchand M, Hainaut P, Pouillart P, Sastre X, Ikeda H, Boon T and Coulie PG (1995) Differences in the antigens recognized by cytolytic T cells on 2 successive mctastases of a melanoma patient are consistent with immune selection. *Eur J Immunol* **25**: 340–347

Lengauer C, Kinzler KW and Vogelstein B (1998) Genetic instabilities in human cancers. *Nature* **396**: 643–649

Ljunggren HG, Stam NJ, Ohlen C, Neefjes JJ, Hoglund P, Heemels MT, Bastin J, Schumacher TNM, Townsend A, Karre K and Ploegh HL (1990) Empty MHC class I molecules come out in the cold. *Nature* **346**: 476–480

Maeda H and Shiraishi A (1996) TGF-beta contributes to the shift toward Th2-type responses through direct and IL-1O-mediated pathways in tumor-bearing mice. *J Immunol* **156**: 73–78

Maeurer MJ, Gollin SM, Martin D, Swaney W, Bryant J, Castelli C, Robbins P, Parmiani G, Storkus WJ and Lotze MT (1996) Tumor escape from immune recognition – Lethal recurrent melanoma in a patient associated with downregulation of the peptide transporter protein TAP-1 and loss of expression of the immunodominant MART-1/Melan-A antigen. *J Clin Invest* **98**: 1633–1641

Marzo AL, Lake RA, Lo D, Sherman L, McWilliam A, Nelson D, Robinson BWS and Scott B (1999) Tumor antigens are constitutively presented in the draining lymph nodes. *J Immunol* **162**: 5838–5845

Matsuda M, Salazar F, Petersson M, Masucci G, Hansson J, Pisa P, Zhang QJ, Masucci MG and Kiessling R (1994) Interleukin-10 pretreatment protects target cells from tumor-specific and allo-specific cytotoxic T cells and downregulates HLA Class I expression. *J Exp Med* **180**: 2371–2376

Medema JP, Toes REM, Scaffidi C, Zheng TS, Flavell RA, Melief CJM, Peter ME, Offringa R and Krammer PH (1997) Cleavage of FLICE (caspase-8) by granzyme B during cytotoxic T lymphocyte-induced apoptosis. *Eur J Immunol* **27**: 3492–3498

Melcher A, Todryk S, Hardwick N, Ford M, Jacobson M and Vile RG (1998) Tumor immunogenicity is determined by the mechanism of cell death via induction of heat shock protein expression. *Nat Med* **4**: 581–587

Morisaki T, Katano M, Ikubo A, Anan K, Nakamura M, Nakamura K, Sato H, Tanaka M and Torisu M (1996) Immunosuppressive cytokines (IL-10, TGF-beta) genes expression in human gastric carcinoma tissues. *J Surg Oncol* **63**: 234–239

Multhoff G, Botzler C, Wiesnet M, Muller E, Meier T, Wilmanns W and Issels RD (1995) A stress inducible 72-Kda heat shock protein (Hsp72) is expressed on the surface of human tumour cells, but not on normal cells. *Int J Cancer* **61**: 272–279

Niehans GA, Brunner T, Frizelle SP, Liston JC, Salerno CT, Knapp DJ, Green DR and Kratzke RA (1997) Human lung carcinomas express Fas ligand. *Cancer Res* **57**: 1007–1012

Ochsenbein AF, Klenerman P, Karrer U, Ludewig B, Pericin M, Hengartner H and Zinkernagel RM (1999) Immune surveillance against a solid tumor fails because of immunological ignorance. *Proc Natl Acad Sci USA* **96**: 2233–2238

O'Connell J, Bennett MW, O'Sullivan GC, Roche D, Kelly J, Collins JK and Shanahan F (1998) Fas ligand expression in primary colon adenocarcinomas: Evidence that the Fas counterattack is a prevalent mechanism of immune evasion in human colon cancer. *J Pathol* **186**: 240–246

Pena JC, Thompson CB, Recant W, Vokes EE and Rudin CM (1999) Bcl-x(L) and Bcl-2 expression in squamous cell carcinoma of the head and neck. *Cancer* **85**: 164–170

Pitti RM, Marsters SA, Lawrence DA, Roy M, Kischkel FC, Dowd P, Huang A, Donahue CJ, Sherwood SW, Baldwin DT, Godowski PJ, Wood WI, Gurney AL, Hillan KJ, Cohen RL, Goddard AD, Botstein D and Ashkenazi A (1998) Genomic amplification of a decoy receptor for Fas ligand in lung and colon cancer. *Nature* **396**: 699–703

Poppas DP, Pavlovich CP, Folkman J, Voest EE, Chen XH, Luster AD and O'Donnell MA (1998) Intravesical bacille Calmette-Guerin induces the antiangiogenic chemokine interferon-inducible protein 10. *Urology* **52**: 268–275

Restifo NP, Esquivel F, Kawakami Y, Yewdell JW, Mule JJ, Rosenberg SA and Bennink JR (1993) Identification of human cancers deficient in antigen processing. *J Exp Med* **177**: 265–272

Romero P, Dunbar PR, Valmori D, Pittet M, Ogg GS, Rimoldi D, Chen JL, Lienard D, Cerottini JC and Cerundolo V (1998) Ex vivo staining of metastatic lymph nodes by class I major histocompatibility complex tetramers reveals high numbers of antigen-experienced tumor-specific cytolytic T lymphocytes. *J Exp Med* **188**: 1641–1650

Rosenberg SA (1999) A new era for cancer immunotherapy based on the genes that encode cancer antigens. *Immunity* **10**: 281–287

Sahin U, Tureci O, Schmitt H, Cochlovius B, Johannes T, Schmits R, Stenner F, Luo GR, Schobert I and Pfreundschuh M (1995) Human neoplasms elicit multiple specific immune responses in the autologous host. *Proc Nat Acad Sci USA* **92**: 11810–11813

Seliger B, Hohne A, Knuth A, Bernhard H, Ehring B, Tampe R and Huber C (1996*a*) Reduced membrane major histocompatibility complex class I density and stability in a subset of human renal cell carcinomas with low TAP and LMP expression. *Clinical Cancer Research* **2**: 1427–1433

*© 2000 Cancer Research Campaign* 

Seliger B, Hohne A, Knuth A, Bernhard H, Meyer T, Tampe R, Momburg F and Huber C (1996*b*) Analysis of the major histocompatibility complex class I antigen presentation machinery in normal and malignant renal cells: Evidence for deficiencies associated with transformation and progression. *Cancer Res* **56**: 1756–1760

Seliger B, Maeurer MJ and Ferrone S (1997) TAP off – Tumors on. *Immunol Today* **18**: 292–299

Strand S, Hofmann WJ, Hug H, Muller M, Otto G, Strand D, Mariani SM, Stremmel W, Krammer PH and Galle PR (1996) Lymphocyte apoptosis induced by CD95 (Apo-1/Fas) ligand expressing tumor cells – a mechanism of immune evasion. *Nat Med* **2**: 1361–1366

Svane IM, Engel AM, Nielsen MB, Ljunggren HG, Rygaard J and Werdelin O (1996) Chemically induced sarcomas from nude mice are more immunogenic than similar sarcomas from congenic normal mice. *Eur J Immunol* **26**: 1844–1850

Taylor Papadimitriou J and Finn OJ (1997) Biology, biochemistry and immunology of carcinoma-associated mucins. *Immunol Today* **18**: 105–107

Todryk S, Melcher AA, Hardwick N, Linardakis E, Bateman A, Colombo MP, Stoppacciaro A and Vile RG (1999) Heat shock protein 70 induced during tumor cell killing induces Th1 cytokines and targets immature dendritic cell precursors to enhance antigen uptake. *J Immunol* **163**: 1398–1408

Torzewski M, Sarbia M, Heep H, Dutkowski P, Willers R and Gabbert HE (1998) Expression of Bcl-X-L, an antiapoptotic member of the Bcl-2 family, in esophageal squamous cell carcinoma. *Clinical Cancer Research* **4**: 577–583

van de Welvankemenade E, Ligtenberg MJL, de Boer AJ, Buijs F, Vos HL, Melief CJM, Hilkens J and Figdor CG (1993) Episialin (MUC1) inhibits cytotoxic lymphocyte-target cell interaction. *J Immunol* **151**: 767–776

Vanky F, Nagy N, Hising C, Sjovall K, Larson B and Klein E (1997) Human ex vivo carcinoma cells produce transforming growth factor beta and thereby can inhibit lymphocyte functions in vitro. *Cancer Immunol Immunother* **43**: 317–323

Wick M, Dubey P, Koeppen H, Siegel CT, Fields PE, Chen LP, Bluestone JA and Schreiber H (1997) Antigenic cancer cells grow progressively in immune hosts without evidence for T cell exhaustion or systemic anergy. *J Exp Med* **186**: 229–238

Yannelli JR, Hyatt C, McConnell S, Hines K, Jacknin L, Parker L, Sanders M and Rosenberg SA (1996) Growth of tumor-infiltrating lymphocytes from human solid cancers: Summary of a 5-year experience. *Int J Cancer* **65**: 413–421

York IA and Rock KL (1996) Antigen processing and presentation by the class I major histocompatibility complex. *Annu Rev Immunol* **14**: 369–396

Young DC (1989) Drug resistance: the clinical problem. In *Drug Resistance in Cancer Therapy* Ozols RF (ed) pp 1–26 Kluwer: Dordrecht

Zhang K, Sikut R and Hansson GC (1997) A MUC1 mucin secreted from a colon carcinoma cell line inhibits target cell lysis by natural killer cells. *Cell Immunol* **176**: 158–165

 *© 2000 Cancer Research Campaign*
